# Manubriosternal Joint Involvement as a Presenting Feature of Axial Spondyloarthritis

**DOI:** 10.7759/cureus.20527

**Published:** 2021-12-20

**Authors:** Melissa Oye, Aleem A Ali, Paul L Wasserman, Gurjit S Kaeley, Myint Thway

**Affiliations:** 1 Internal Medicine, University of Florida College of Medicine – Jacksonville, Jacksonville, USA; 2 Radiology, University of Florida College of Medicine – Jacksonville, Jacksonville, USA; 3 Rheumatology, University of Florida College of Medicine – Jacksonville, Jacksonville, USA

**Keywords:** inflammatory disease, hla-b27, arthritis, chest wall pain, spondyloarthritis

## Abstract

Anterior chest wall pain is a feature of axial spondyloarthritis that is understudied. It is rarely the presenting symptom, and when present, may suggest severe disease. We present the case of a 35-year-old female with recurring presentations of debilitating chest pain, subsequently diagnosed with axial spondyloarthritis. Awareness of this presentation can lead to earlier diagnosis and treatment of patients presenting with manubriosternal joint involvement as their initial symptom of axial spondyloarthritis.

## Introduction

Spondyloarthritis is a spectrum of chronic inflammatory disease affecting the axial skeleton, peripheral joints, entheses, and other organ systems. It is divided into categories based on the predominant clinical features. Axial spondyloarthritis (AxSpA) is the prototype of immune-mediated inflammatory rheumatic diseases mainly with the involvement of the axial system, especially sacroiliac joint involvement, strongly linked with HLAB27.

Inflammatory back pain is the entry symptom in diagnosing AxSpA. Diagnosis depends on the level of certainty, whereas classification criteria are dichotomous. Features used by classification criteria are useful for diagnostic purposes. Earlier classification criteria such as the Modified New York Criteria emphasized radiologic sacroiliitis changes. More recent classification criteria such as the Assessment of Spondyloarthritis International Society allow non-radiographic features to be included. However, atypical or rarer features are not included in these classification criteria [1]. Recent studies showed that early diagnosis is essential to have a better therapeutic outcome and can preserve functional status [2].

## Case presentation

A 35-year-old Caucasian female with prior history of anxiety presented with progressively worsening sternal chest pain that radiated to bilateral shoulders over the previous five months. Initially, the pain was associated with deep inspiration and movement; however, it became constant throughout the day. The patient was unable to work, exercise, or perform activities of daily living. There was no reported history of trauma or inciting factors.

Initial workup by her primary care physician with chest radiograph, electrocardiogram, and chest computed tomography (CT) was unrevealing. Lab investigations revealed complete blood count (CBC) with elevated platelets (451,000/µL), chemistry panel was within normal limits, and negative cardiac biomarkers. As a cardiopulmonary etiology was excluded, the patient’s pain was thought to be musculoskeletal in nature, rather than pleuropericardiac. She was diagnosed with costochondritis and prescribed a short course of nonsteroidal anti-inflammatory drugs (NSAIDs), corticosteroids, and topical lidocaine.

**Figure 1:**
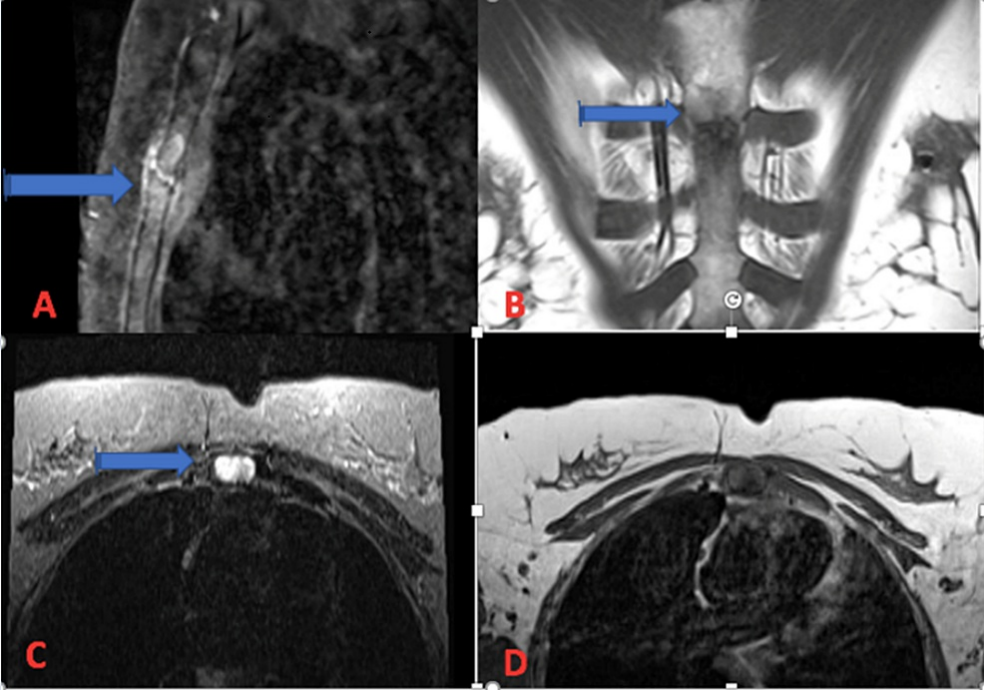
(A) Sagittal T1 fat-saturated post-contrast image of manubriosternal joint showing abnormal signal and enhancement and small erosions. (B) Coronal T1 MRI of manubriosternal joint. (C) Axial T2/STIR MRI of manubriosternal joint showing small erosions. (D) Axial T1 MRI of manubriosternal joint.

Despite the above therapy, the patient’s chest wall pain persisted, prompting her to seek consultation with a rheumatology specialist. On rheumatology review, the patient had exquisite chest wall pain and tenderness over the manubrium and body of sternum on palpation with labs revealing a normal erythrocyte sedimentation rate (ESR) and C-reactive protein (CRP) with reactive thrombocytosis. Due to the severity of her symptoms, multi-modal imaging of the chest wall was pursued. Magnetic resonance imaging (MRI) chest revealed an eroded sternomanubrial joint with abnormal periarticular marrow signal and enhancement, representing inflammatory arthritis (Figures 1A-1D). The patient later endorsed a history of lower back pain. MR images of the pelvis revealed erosions and sclerosis of the sacroiliac joints bilaterally, suggestive of inflammatory arthritis. She was found to have a positive HLA B27. Diagnosis of AxSpA was made based on positive HLAB27, and abnormal MRI with joint erosions. The patient was able to be treated with a tumor necrosis factor (TNF) alpha inhibitor with improvement in symptoms.

## Discussion

AxSpA is chronic inflammatory arthritis affecting the axial skeleton and sacroiliac joints. This condition affects less than 1% of the population [3]. Inflammatory back pain is often the presenting symptom of spondyloarthritis, with most patients reporting anterior chest wall pain after the onset of back pain [4].

Manubriosternal joint involvement causing anterior chest wall pain is rarely seen as the presenting symptom of AxSpA; however, it is more commonly reported in women [4]. In a retrospective study performed in 275 patients with spondyloarthritis, 37% of these patients experienced spondyloarthritis-associated chest wall pain [5]. However, anterior chest wall pain as the presenting symptom occurs in only 4%-6% of cases [6]. Anterior chest wall pain occurs in 30%-60% of patients with AxSpA, presenting both in the earlier and later stages of the disease process [7].

High clinical suspicion and consideration of a wide differential diagnosis are paramount in patients presenting with non-cardiac, atypical anterior chest wall pain. Some diagnoses may be more apparent, such as a sternal fracture or dislocation secondary to trauma, but others are more discrete. Arthritides that can affect the manubriosternal joint include osteoarthritis, rheumatoid arthritis, septic arthritis, and seronegative arthropathies [8]. Differentiation between the arthritides is important for proper treatment and a detailed history and physical exam can often provide vital clues. Primary septic arthritis of the manubriosternal joint is a rare entity with a handful of cases reported in the literature [8-11]. Pyrexia and elevated inflammatory markers are often observed [8]. Associated predisposing factors include immunodeficiency, underlying joint disease, hemoglobinopathy, and intravenous drug use [8,12]. Malignancy should also be considered. While primary sternal tumors are uncommon, secondary involvement from metastases can be seen in a variety of solid organ and hematologic cancers [12].

The anterior chest wall pain of AxSpA is due to diffuse or localized enthesitis of the sternocostal, sternoclavicular, and/or the manubriosternal areas described as an acute, sharp pain exacerbated with upper extremity and respiratory movements. Sternoclavicular joint involvement has been reported in 17%-50% of affected patients with manubriosternal joint involvement in 51% of patients [6].

Diagnosis of AxSpA requires evidence of sacroiliitis on radiologic imaging, inflammatory symptoms suggestive of spondyloarthritis, with some patients being HLA-B27 positive. MRI can detect early changes in spondyloarthritis, revealing subtle erosions, bone edema, and signs of inflammation. Another hallmark feature of AxSpA that has been well documented is structural lesions with new bone formation [13]. This can be assessed on MRI, and early signs of bone formation can help lead to the diagnosis of AxSpa. This bone formation can form not only in the sacroiliac joints but also in the manubriosternal joint potentially impacting chest wall movement. Findings of bone formation can also be helpful in monitoring disease progression [13]. Early evaluation and diagnosis of spondyloarthritis can lead to earlier initiation of treatment with both pharmacologic and nonpharmacologic interventions.

## Conclusions

Anterior chest wall pain in the setting of manubriosternal joint involvement can be the presenting symptom in patients with AxSpA; however, it is rare and a high index of suspicion is required. AxSpA diagnosis in females is delayed usually because of the atypical presentation of chest wall involvement. Inflammatory markers may not be elevated in most patients with AxSpA. MRI of the chest wall is the best modality to assess for signs of inflammatory arthritis when plain X-ray imaging is negative. Treatment with TNF alpha inhibitors is preferred in patients who fail treatment with NSAIDs.
